# Methodological concerns regarding supraphysiological histidine concentrations in HCC immunometabolism studies: an evidence-based discussion

**DOI:** 10.1186/s12967-026-07928-2

**Published:** 2026-03-04

**Authors:** Chenyang Ma, Xiaofei Zhu

**Affiliations:** 1 Department of Science and Education, Hangzhou Xiaoshan Second People’s Hospital, Hangzhou, China; 2 Department of Infectious Diseases, Hangzhou Ninth Hospital, Hangzhou, Zhejiang Province China

To the Editor: 

We commend Liu et al. for their exploration of immunometabolism in hepatocellular carcinoma (HCC) in the article “*Histidine metabolism drives liver cancer progression via immune microenvironment modulation through metabolic reprogramming*” in Journal of Translational Medicine [1]. However, we have significant methodological concerns about the use of a 30 mM histidine concentration in their in vitro models, which may critically affect the interpretation and translational potential of their findings.

Firstly, the employed histidine concentration utilized in this study, set at 30 mM (30,000 µM), significantly surpasses physiological levels. The typical physiological plasma concentration of histidine in humans is approximately 70 µM [2]. While no specific guidelines were found that mandate amino acid concentrations to be ≤ 1 mM, it is a widely recognized principle in cell culture research to employ physiologically relevant concentrations in order to mitigate the risk of obtaining artifactual results [3]. The application of concentrations that are 430-fold greater than physiological levels is likely to induce osmotic stress and non-physiological metabolic alterations, and may even lead to off-target cytotoxic effects.

Moreover, while it could be argued that high concentrations may enhance signal detection, the absence of concentration-gradient experiments precludes a clear differentiation between specific immunomodulatory effects mediated by histidine metabolism [4] and nonspecific artifacts arising from extreme hyperosmolarity or cytotoxicity. As highlighted in their Methods section, the lack of comprehensive viability assays specifically for cells treated with 30 mM histidine [1] further complicates the determination of the mechanistic specificity of the observed effects. To preserve the valuable mechanistic insights of this study, we strongly recommend that the authors replicate key experiments using a concentration-response design within physiological ranges. This approach has been successfully employed in previous HCC metabolomics research [5].

In conclusion, the application of 30 mM histidine, although empirically validated in contexts unrelated to histidine metabolism, becomes fundamentally problematic when employed in studies focused on histidine’s metabolic role in HCC. This methodological discrepancy poses a risk of producing mechanistically misleading conclusions, as the excessively high concentration is likely to induce non-physiological artifacts that obscure the authentic metabolic functions. Consequently, employing a pathologically relevant histidine concentrations gradient is fundamental to paving the way for a rigorous understanding of its metabolic functions in HCC.

## Data Availability

Not applicable.
